# Is it time to use nucleic acid amplification tests for identification of persons with sexually transmitted infections?: evidence from seroprevalence and behavioral epidemiology risk surveys in men with chlamydia and gonorrhea

**DOI:** 10.11604/pamj.2020.36.299.20777

**Published:** 2020-08-18

**Authors:** Laura Tobin, Lydia Guerra, Léonce Ahouanvoeke, Jose González Carpio, Donatien Irambona, Edward Owusu Nyarko, Caroline Macera, Steven Wiersma

**Affiliations:** 1 Department of Defense, HIV/AIDS Prevention Program, Combat Support Agency, Defense Health Agency, United States Department of Defense, San Diego, California, United States of America,; 2 Belize Defense Force, Belize City, Belize,; 3 Benin Army Health System, Cotonou, Benin,; 4 Dominican Republic Air Force (FARD), Santo Domingo, Dominican Republic,; 5 Office of the Minister of National Defense and Veterans Affairs, Burundi National Defense Force, Gitega, Burundi,; 6 Public Health Division, Ghana Armed Forces, Accra, Ghana

**Keywords:** Sexually transmitted infection, syndromic diagnosis, nucleic-acid amplification test

## Abstract

Chlamydia and gonorrhea are common sexually transmitted infections (STIs) that can cause multiple problems, and can be easily treated, but frequently present without symptoms. Because of this, commonly used syndromic diagnosis misses a majority of infected persons. Previously, diagnostic tests were expensive and invasive, but newer nucleic-acid amplification tests (NAATs) are available that use urine to non-invasively test for these infections. These analyses used data from seroprevalence studies conducted in five militaries. Data included self-reported current symptoms of STIs as well as chlamydia and gonorrhea NAAT results. A total of 4923 men were screened for chlamydia and gonorrhea from these 5 militaries during April 2016 to October 2017. The combined prevalence of chlamydia and gonorrhea in these five militaries ranged from 2.3% in Burundi to 11.9% in Belize. These infections were not successfully identified by symptomology; for example, only 2% of cases in Belize reported symptoms. In three of the five countries there was no statistical association between symptoms and positive NAAT results. The majority of individuals with these infections (81% to 98%) would be undiagnosed and untreated using only symptomology. Therefore, using symptoms alone to diagnose cases of chlamydia and gonorrhea is not an effective way to control these infections. We propose that automated, cartridge-based NAATs, be considered for routine use in diagnosing those at risk for STIs.

## Introduction

Sexually transmitted infections (STIs) cause significant burden of disease worldwide [1]. The complications of curable STIs that are left untreated include pelvic inflammatory disease, ectopic pregnancy, infertility, chronic pelvic pain, arthropathy, neurological and cardiovascular diseases. Many STIs, including chlamydia (CT) and gonorrhea (NG), also put infected individuals at increased risk for acquiring and transmitting human immunodeficiency virus (HIV) [2]. In many countries, health systems use symptoms to identify persons with suspected STIs. For cases of CT and NG, this approach focuses on symptoms including painful urination and penile or vaginal discharge. According to a 2015 World Health Organization (WHO) assessment, 96% of countries in the WHO Africa region employed syndromic STI management, while only 8.5% attempted to determine laboratory-based etiology of symptoms [3]. The majority of people infected with CT/NG are asymptomatic. These persons may or may not seek health care for other reasons [4]. However, identifying and treating asymptomatic persons can prevent the development of STI-related complications for the individual and help reduce the transmission in the community. Nucleic acid amplification tests (NAAT) employ molecular-based technology to identify the presence of the *Neisseria gonorrhea and Chlamydia trachomatis* the causative organisms of gonorrhea and chlamydia. Before the development of NAATs to diagnose CT and NG infections, testing for STIs required invasive specimen collection by healthcare professionals. Now, NAAT technology uses urine specimens provided by clients, eliminating the need for an examination from a healthcare provider. NAATs also have increased specificity and sensitivity compared to other methods and are often able to detect infection even when there is only a small number of bacteria present [5]. National, regional, and global estimation of STI prevalence and incidence rely on prevalence surveys among general populations. More often STI prevalence surveys are conducted among women as compared to men [6,7]. This analysis provides valuable methods and STI prevalence data for male military populations in these countries which can be used to estimate national and regional STI burden. This prevalence study compared the efficacy of the symptomatic screening method utilized by the health systems of military medical services in select Latin American and African countries with gold-standard NAAT for the diagnosis of CT and NG infections. The purpose was to determine if the symptomatic approach is sufficient to identify individuals in need of treatment for CT and NG infections in low resource settings.

## Methods

These secondary analyses used survey and NAAT laboratory screening data for chlamydia and gonorrhea from five cross-sectional Seroprevalence and Behavioral Epidemiology Risk Surveys (SABERS) studies conducted in the militaries of Belize, Benin, Burundi, Dominican Republic, and Ghana [8]. All studies were approved by both local and US-based Institutional Review Boards (IRBs). To be included in the SABERS studies, individuals had to be 18 years of age or older, currently on active military duty, and stationed at a site selected for study inclusion. Due to the low number of female participants as well as symptom and diagnostic test performance differences, these secondary analyses included only consenting male participants. Men who were missing symptom or diagnostic data were excluded. The final samples sizes are provided in Table 1. All data were collected electronically on tablets. Symptoms were assessed using a self-report survey item that identified those with current pain during urination or abnormal penile discharge. CT and NG diagnoses were determined using NAAT analysis of urine samples. The prevalence values displayed in Table 1 represent the combined prevalence of both CT and NG infections. Individual and co-infection prevalence values are presented in Table 2. Sensitivity, specificity, positive predictive value (PPV), and negative predictive value (NPV) were calculated using standard formulas [9]. True positives were defined as individuals with a positive CT/NG NAAT result who also reported one or more symptoms. True negatives were defined as individuals with a negative CT/NG NAAT result and no reported symptoms. Logistic regression was used to calculate an unadjusted odds ratio to describe the statistical association between reporting current symptoms and a positive CT/NG NAAT result.

**Table 1:** performance of symptom-based diagnosis of male CT/NG infection

	Belize	Benin	Burundi	Dominican Republic	Ghana
**N**	412	1242	1133	1216	920
**Collection dates**	Mar-Apr 2017	Aug-Sep 2017	Sep-Oct 2017	May-Jun 2017	Apr-May 2016
**Median age (range)**	27 (18-53)	31 (21-50)	38 (20-55)	29 (18-76)	31 (19-65)
**Prevalence of current STI symptoms (n)**	3.4% (14)	6.4% (79)	14.8% (168)	3.0% (37)	2.8% (26)
**True CT/NG prevalence (n)**	11.9% (49)	7.3% (91)	2.3% (26)	7.5% (91)	8.9% (82)
**PPV**	7.1%	21.5%	2.4%	13.5%	30.8%
**NPV**	87.9%	93.6%	97.7%	92.7%	91.7%
**Sensitivity**	2.0%	18.7%	15.4%	5.5%	9.8%
**Specificity**	96.4%	94.6%	85.2%	97.2%	97.9%

**Table 2:** prevalence of NAAT diagnosed CT, NG, and CT/NG co-infection

Country	N	CT prevalence (n)*	NG prevalence (n)*	CT/NG co-infection prevalence (n)
**Belize**	412	11.4% (47)	1.0% (4)	0.5% (2)
**Benin**	1242	6.8% (85)	0.5% (6)	0 (0)
**Burundi**	1133	1.2% (14)	1.3% (15)	0.3% (3)
**Dominican Republic**	1216	7.2% (88)	0.5% (5)	0.2% (2)
**Ghana**	920	7.9% (73)	1.0% (9)	0 (0)

* Includes co-infected individuals

## Results

The combined CT/NG prevalence of each military ranged from 2.3% (26/1133) in Burundi to 11.9% (49/412) in Belize (Table 1). Syndromic screening functioned poorly to identify CT/NG positive individuals. Using validated NAAT technology as a reference standard, the sensitivity of symptom-based CT/NG diagnosis was lowest in Belize, where only 2.0% (1/49) of positives were identified with symptoms. Symptom-based sensitivity was highest in Benin, identifying 18.7% (17/74) of positives. The PPV ranged from 2.4% in Burundi (2.3% prevalence) to 30.8% in Ghana (8.9% prevalence), indicating that the majority of people with symptoms did not have a CT or NG infection. The NPV was a better predictor of negative status. The highest NPV was in Ghana (97.9%) and as expected, the NPV was lowest in Burundi (85.2%), the country with the lowest prevalence. In addition to calculating performance metrics, logistic regression was used to examine the unadjusted statistical association between reporting current symptoms and testing positive for CT/NG using NAAT (Table 3). In the Dominican Republic, Burundi, and Belize, there was no association between having symptoms and a positive NG/CT NAAT result. However, in Benin, symptomatic individuals were four times more likely to test positive for NG/CT compared to asymptomatic individuals. In Ghana, the magnitude of the association was even greater, as symptomatic individuals were five times more likely to test positive compared to those without symptoms.

**Table 3:** associations between reporting current symptoms and NAAT positivity for CT/NG infection

Country	Odds ratio	95% CI	p-value
**Belize**	0.56	0.07-4.38	0.58
**Benin**	4.04	2.25-7.25	<0.01
**Burundi**	1.05	0.36-3.07	0.94
**Dominican Republic**	1.63	0.73-3.67	0.23
**Ghana**	4.93	2.07-11.71	<0.01

## Discussion

Overall, symptoms are a poor indicator of CT/NG infection. In three of the five countries, there was no statistically significant association between being symptomatic and testing positive for CT/NG using NAAT. Additionally, using this method left the majority of CT/NG positive individuals (81-98%) undiagnosed and untreated. Of those who did present with symptoms, only 2-31% truly had a CT/NG infection. Additionally, symptom only screening did not function uniformly across the five country militaries. A limitation of this study was that STI screening was limited to CT/NG and it is possible that symptomatic individuals had another infection or a non-infectious etiology. However, we believe that CT/NG is a major cause of urethral syndrome in these settings. Additionally, NAAT was performed in different labs on a variety of systems which could have added some inconsistency to the final CT/NG test results. However, the published manufacturer sensitivities and specificities of the NAAT methods that were used ranged from 98.0-98.5 (sensitivity) and 99.6-99.8 (specificity) for NG and 97.5-98.5 (sensitivity) and 98.4-99.4 (specificity) for CT. Therefore, it is unlikely that this accounts for all the performance variability observed in these results.

## Conclusion

Using symptoms alone to identify individuals for CT/NG treatment is not an effective approach to control these infections. Automated, cartridge-based nucleic amplification assays are becoming increasingly available in low-resource settings [10]. These have been deployed largely for tuberculosis and HIV viral load testing but also offer the ability to test for STIs including CT/NG. Due to the large burden of disease and poor performance of syndromic screening for STIs, it is time to consider alternate strategies for identifying who would benefit from prompt diagnosis and treatment and to reduce the transmission of STIs. Further studies are needed to address costs and cost-effectiveness of NAAT-based approaches to STI control. STI prevalence surveys can be conducted alongside those of other biobehavioral surveys to improve STI surveillance and burden estimation among general and at risk populations [11].

### What is known about this topic

- Asymptomatic infection is common with Chlamydia trachomatis and Neisseria gonorrhoeae;
- Many low-resource countries typically treat sexually transmitted infections based on symptoms alone, even though symptoms are rarely present with these infections.

### What this study adds

- This multi-national prevalence survey of chlamydia and gonorrhea among male military personnel identified high rates of asymptomatic infection;
- Relatively inexpensive, non-invasive testing is available to diagnose chlamydia and gonorrhea. If these tests were used in lieu of the symptom only approach, significantly more individuals would be identified and treated;
- STI prevalence surveys can be conducted alongside those of other biobehavioral surveys to improve STI surveillance and burden estimation among general and at-risk populations.
